# Comparison of different automatic methods for the delineation of the total metabolic tumor volume in I–II stage Hodgkin Lymphoma

**DOI:** 10.1038/s41598-020-69577-9

**Published:** 2020-07-28

**Authors:** Queralt Martín-Saladich, Gabriel Reynés-Llompart, Aida Sabaté-Llobera, Azahara Palomar-Muñoz, Eva Domingo-Domènech, Montse Cortés-Romera

**Affiliations:** 1 Medical Physics Department, Institut Català d’Oncologia, L’Hospitalet de Llobregat, Barcelona, Spain; 2 PET Unit, Nuclear Medicine Department, IDI, Hospital U. de Bellvitge-IDIBELL, L’Hospitalet de Llobregat, Barcelona, Spain; 3 Hematology Department, Institut Català d’Oncologia, L’Hospitalet de Llobregat, Barcelona, Spain

**Keywords:** Molecular medicine, Oncology

## Abstract

Total metabolic tumor volume (TMTV) is a promising quantitative biomarker for therapy assessment and prognosis in Hodgkin Lymphoma affected patients that allows prediction of patient outcome. The aim of this study was to evaluate the TMTV reproducibility between different sources of variability in tumor delimitation such as SUV-based thresholds (2.5, 41% and 50%) and software tools (Beth Israel plugin (BI) and LIFEx). Effect of contouring procedure both including single and multiple regions of interest was also studied in patients with multiple lesions, and optimal cut-offs for each studied method were displayed to compare the effect on prognosis. Strong alikeness in TMTV was found for 2.5 under software choice*.* Best accuracy in contouring compared to visual assessment of the disease was found for BI multiple ROI and LIFEx single ROI drawing. Similar cut-offs were found between both software for all considered thresholds, but best resemblance and highest cut-off due to an overestimation of the TMTV was found for 2.5 SUV. Our findings suggest that optimal reproducibility in TMTV is found for SUV > 2.5 threshold under choice of contouring methodology or software tool, meaning that overestimation of the TMTV threshold using 2.5 looks to be preferable than underestimation with 41% and 50%.

## Introduction

[^18^F]FDG PET/CT imaging has been used to assess the total metabolic tumor volume (TMTV) in the clinical staging of newly diagnosed Hodgkin lymphoma^1^. However, false-positive results have led to the use of new methods to face non-specificity of [^18^F]FDG uptake and the lack of differentiation between malignancy and physiological background. Many methodologies on how to estimate TMTV have been recently proposed but no general agreement has been achieved. Most of the latterly published studies have based their method on defining thresholds that act as a discriminant for TMTV^2^, some of which are based on standardized uptake value (SUV) obtained in [^18^F]FDG PET/CT^3^. Both absolute and relative thresholds are frequently used, although more complex algorithms including iterative thresholds that correct partial-volume effect (PVE), and adaptive cut-offs based on lesion-to-background ratio are also being employed^4^.

Finding a common unified method is necessary for forthcoming studies which could allow comparisons between institutions leading to optimal patient management within medical centers^5^. Software tools can be a source of discrepancy between obtained TMTV values due to internal segmentation algorithms. However, threshold choice within the software has been proven to influence the TMTV, though no significant differences between predicted prognosis have been found^6^. Therefore, contrasting the effect of software packages and different thresholds on the TMTV estimation could help predict future patients outcome, basing their survival foreshadow on the parameters given by the chosen methodology^7^. Despite the availability of advanced algorithms for the delimitation of the TMTV, the only universal multivendor methods are the simple ones based on defined thresholds, as recommended for a general oncological case in the EANM 2.0 protocol^8^.

The aim of this study was to delimit TMTV in Hodgkin lymphoma patients (Ann-Arbor stage I and II) on baseline [^18^F]FDG PET/CT acquisitions. Contouring was performed using different software tools (BI and LIFEx^6,9^) and TMTV discrimination was achieved using SUV based thresholds (2.5 SUV, 41% SUV_max_, and 50% SUV_max_). Both potential sources of bias were analyzed to compare TMTV reproducibility. The effect of multiple and single ROI drawing procedures in multiple lesion affected patients was also studied. Research also focused on the effect of evaluated parameters on the prognosis of the disease to predict patient outcome.

## Material and methods

This manuscript has been revised for its publication by the Clinical Research Ethics Committee of Bellvitge University Hospital. Written informed consent was waived by this Committee, as it was a retrospective analysis of our usual everyday work. The data of the patients were anonymized for the purposes of this analysis. The confidential information of the patients was protected according national normative.

### Patients’ characteristics and image acquisition

Out of a database of 116 HL affected patients within our institution, only 76 were compared and analyzed. The 40 remaining were excluded due to DICOM loading failure (6), Ann-Arbor stages III–IV (12), interim or post excision (4), missing data in image acquisition (4) and under nuclear medicine specialist criteria (14), which included no follow up (3), relapse (5), and misclassified patients (6). Patient characteristics including bulky mass for lesions such > 10 cm and histologic subtypes are shown in Table 1. The most frequent treatment in the evaluated patients was ABVDx4 ± RT, however, the therapies varied after deciding the therapeutic plan in a multi-disciplinary committee according to clinical characteristics, baseline risk factors, the interim PET study and also patients' tolerance to chemotherapy. Those included ABVDx2 ± RT, ABVDx3 ± RT, ABVDx6 ± RT, ABVDx8 ± RT and some even escalated to BEACOPP. Due to the retrospective nature of the study and the non-homogeneity of the treatments administered, with a multitude of subgroups, the analysis of these data has not been performed. PET/CT images were acquired in a Discovery ST (D-ST) or Discovery IQ (D-IQ) PET/CT (GE Healthcare, Waukesha, WI) at 70 ± 15 min after intravenous administration of 2.3–3.7 MBq/kg of [18F]FDG, being mandatory to have a blood glucose level below 10 mmol/L at the time of the radiopharmaceutical injection. The scanning protocol included a torso imaging (from the skull base to mid-thigh), with arms raised whenever possible. Studies from D-ST were reconstructed with a VPHD algorithm with 21 subsets and 2 iterations, and a 6.0 mm Gaussian filter, using a matrix of 128 × 128. D-IQ images were reconstructed with a VPHD-S (VPHD with a point-spread function correction) algorithm with 12 subsets and 4 iterations, and a 4.8 mm Gaussian filter, using a matrix of 256 × 256. Effect of distinct spatial resolution will be minimum for localized lymphoma as tumors are big enough. The effect of scanner choice was studied contrasting potential differences between D-ST and D-IQ scanners using a paired *t* test before mixing both samples.

**Table 1:** Patients’ characteristics.

	Mean	Median	Range
Age (years)	37.5	35	16–87
Follow-up (months)	49.9	51.5	3–104

	Number of patients	Proportion (%)	
**Characteristics**			
Female	39	51.3	
Male	37	48.7	
D-ST PET/CT	69	90.8	
D-IQ PET/CT	7	9.2	
Ann-Arbor stage I	6	7.9	
Ann-Arbor stage II	70	92.1	
Bulky mass	7	9.2	
Mediastinal	15	19.7	
**Histological subtypes**			
Nodular sclerosis	54	71.0	
Mixed cellularity	10	13.2	
Lymphocyte rich	2	2.6	
Lymphocyte depletion	10	13.2	

### Determination of TMTVs

**Figure 1:**
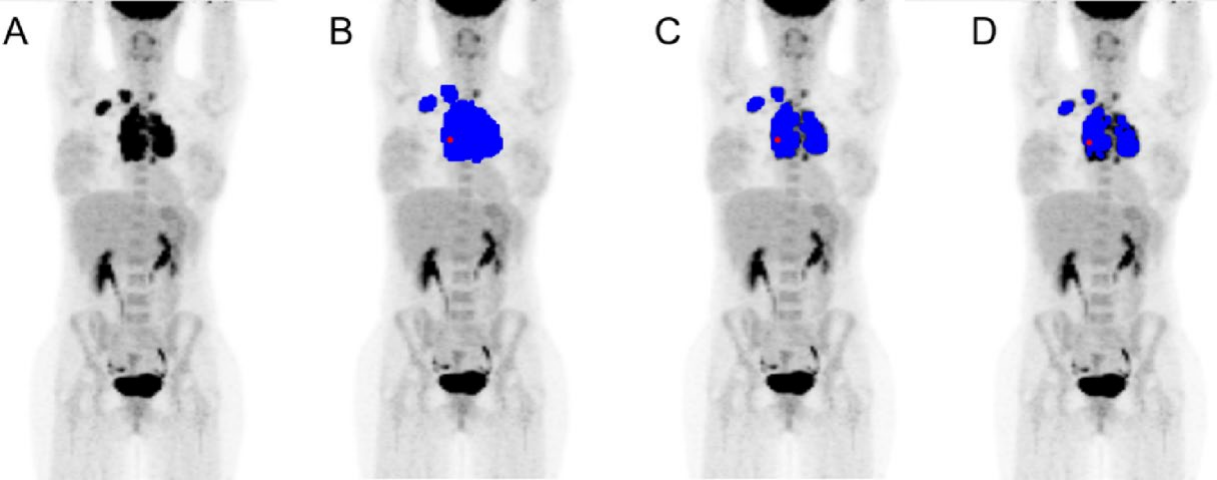
Threshold comparison using BI, (**A**) baseline, (**B**) 2.5 SUV, (**C**) 41% SUV_max_ and (**D**) 50% SUV_max_.

The TMTV were delimited in both software by a medical physicist and supervised and approved by a specialist nuclear medicine physician. Multiple physician supervision was not considered essential due to the use of automatic methods for localized lesions. To delimit TMTV with the BI software using a single region of interest (ROI), a ROI involving all focuses of [^18^F]FDG activity was drawn in one of the slices of each patient avoiding physiological uptake. Despite the difficulty of this procedure, since in many of the patients the injured region was at a different depth in the frontal axis than the physiological uptakes from the heart or lymph nodes, we were able to perform the analysis with precision by the definition of the single ROI in most of the cases. It was then extrapolated to the limiting slices obtaining a volume of interest (VOI) to which 2.5, 41% and 50% cut-offs were applied (Fig. 1). The software automatically selected and highlighted the voxels with a SUV value over the threshold and calculated the TMTV, SUV_mean_, SUV_max_, and SUV_peak_. Total lesion glycolysis (TLG) was later calculated for each threshold as the product of SUV_mean_ and TMTV. The procedure for LIFEx was performed similarly but the ROIs were modified slice by slice, detecting only tumoral regions and avoiding physiological uptake healthy organs. The mentioned thresholds were applied to the defined VOI and the same parameters were calculated.

There are two different approaches to segment the TMTV. A single ROI covering all avid regions can be drawn as explained in the previous paragraph, but there is a second and more laborious approach consisting in performing individual multiple ROIs for each lesion. Using only the BI software we studied the effect of both different methodologies in the definition of the TMTV for those patients who had multiple lesions.

### Data and statistical analysis

For the study and comparison of TMTV values between software and thresholds, as well as for TLG and SUV_mean_ values, due to non-normal distributions data were log-transformed. The statistical analysis and graphs were done using those results. TMTV and TLG values were expressed quantitatively as their mean and their median, specifying the range of values within the data set.

TMTV, SUV_mean_, TLG, SUV_max_, and SUV_peak_ values were analyzed for both software using the mentioned relative and absolute thresholds. Latter comparisons were made. Multiple and single ROI drawing consequences on volume contouring were studied and compared between software tools and thresholds.

Differences and correlation between the two studied samples were displayed using Bland–Altman analysis and Pearson coefficient respectively, and their corresponding distributions of data using box plots showing the p-value were obtained in the paired sample double tailed *t* test for all the parameters mentioned above (TMTV, SUV_mean_, TLG, SUV_max_, and SUV_peak_).

Study of the effect of PET machinery was analyzed using the independent (non-paired) sample *t* test applied to the previous normalized data of TMTV using logarithms. Comparison was performed according if imaging was acquired with D-ST or D-IQ.

Progression-free survival (PFS) was also drawn including both deaths and lymphoma progression. For both software packages, survival curves using the Kaplan–Meier estimator were calculated for each threshold comparing two subgroups split by the TMTV cut-off found in the Receiver Operating Characteristics (ROC) curve. This value was determined by the Youden index according to the optimal sensitivity and specificity based on the comparison of two related TMTV samples. ROC curves described the evolution of HL patients and predicted treatment failure in terms of the Area Under the Curve (AUC). For software comparison, ROC curves were also drawn using the same threshold to show the differences between both TMTV cut-offs and to contrast HL progression.

## Results

Comparison between both PET machines D-ST and D-IQ showed no statistical significance in TMTV values neither for BI nor LIFEx (p = 0.26 and p = 0.27 for 2.5, p = 0.07 and p = 0.16 for 41%, and p = 0.09 and p = 0.23 for 50%, respectively). All SUV_max_ values were exactly the same when comparing software packages and thresholds in all of the studied cases. SUV_peak_ values between different thresholds within BI were statistically significant (p < 0.02) and for LIFEx exact coincidence could be observed. For 2.5 and 41% thresholds the comparison between BI and LIFEx showed significant differences (p < 0.0001) while for 50% less differences could be seen (p = 0.22).

### TMTV

**Figure 2:**
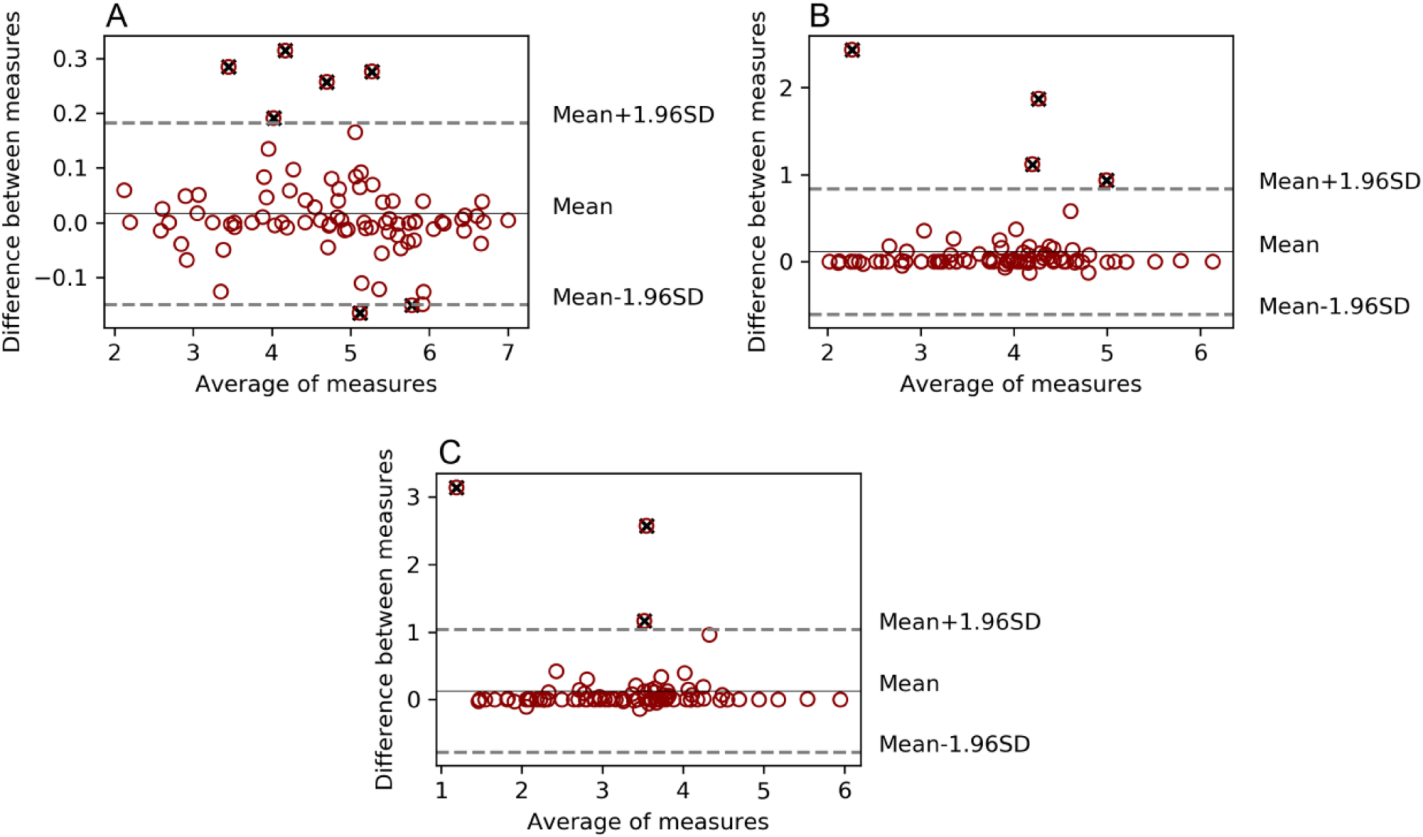
Bland Altman analysis for TMTV obtained using different thresholds. Comparison between software tools BI versus LIFEx. SUV > 2.5 threshold delimitation (**A**), SUV > 41% SUVmax (**B**) and SUV > 50% SUVmax (**C**). Values outside the agreement region in dashed grey lines were marked as black cross outliers but were not discarded from the analysis.

Statistical significance could be observed between TMTV obtained using different thresholds (TMTV_2.5_, TMTV_41%_, and TMTV_50%_) within the same software (p < 10^–20^), as well as when comparing both software applying the same threshold for 41% and 50% (p < 0.02). However, fewer differences could be found in 2.5 (p = 0.09). A strong correlation was obtained in the comparison of 41% and 50% TMTV (r > 0.97) for both software tools, and when comparing BI with LIFEx for the different thresholds TMTV_2.5_ (r = 0.997), TMTV_41%_ (r = 0.92) and TMTV_50%_ (r = 0.89), as shown in the respective Bland Altman analyses (Fig. 2).

Outliers displayed in the previous graph were acquired as it follows: 7 outliers (6 ST-AC and 1 IQ-AC) for the 2.5 threshold, 4 outliers (3 ST-AC and 1 IQ-Q.Clear) for the 41%, and 3 outliers (ST-AC) for the 50%. Obtained values performing the study considering the whole set of patients and using BI and LIFEx as contouring software are displayed in Table 2.

**Table 2:** Study for the whole set of patients without considering the number of lesions they had. Contouring performed using BI and LIFEx. P values compared the mentioned pair of data.

Threshold	Study type	BI (cm^3^)			LIFEx (cm^3^)			p value
		Mean	Median	Range	Mean	Median	Range	BI vs LIFEx
2.5	Whole set	208.46	137.11	9–1,102	208.45	139.10	8–1,038	0.09
41%	Whole set	69.31	52.71	8–461	62.24	50.66	3–462	0.006
50%	Whole set	44.29	31.20	4–384	40.00	27.87	1–384	0.001

### Multiple ROI study

This study was performed with those patients who had multiple affected regions within their body. For this concrete set of patients, when contrasting the effect of drawing single or multiple ROIs in BI compared to the TMTV obtained in LIFEx single ROI drawing, more similarities to LIFEx were found when using BI multiple ROIs for 2.5 (r = 0.995, p = 0.33) than when using single ones (r = 0.997, p = 0.09) as shown in Table 3. Nonetheless, for 41% and 50% statistical significance was found in the use of single ROIs (r = 0.92, p = 0.006 and r = 0.89, p = 0.013), as well as when using multiple ones (r = 0.92, p = 0.0004 and r = 0.96, p < 10^–5^).

**Table 3:** Single ROI study vs multiple ROI study. For the multiple lesion affected patients, values obtained in multiple ROI drawing in BI were compared to the ones obtained in single ROI drawing in LIFEx. P values compared the mentioned pair of data.

Threshold	Study type	BI (cm^3^)			LIFEx (cm^3^)			p value
		Mean	Median	Range	Mean	Median	Range	BI vs LIFEx
2.5	Single ROI	250.96	203.41	9–805	249.84	189.80	9–804	0.11
	Multiple ROI	247.85	197.74	9–794	–	–	–	0.07
41%	Single ROI	96.21	75.90	13–461	86.66	72.08	13–462	0.004
	Multiple ROI	101.90	76.52	17–470	–	–	–	0.04
50%	Single ROI	63.10	44.20	8–384	57.82	43.62	8–384	0.004
	Multiple ROI	66.06	47.14	11–393	–	–	–	0.007

**Figure 3:**
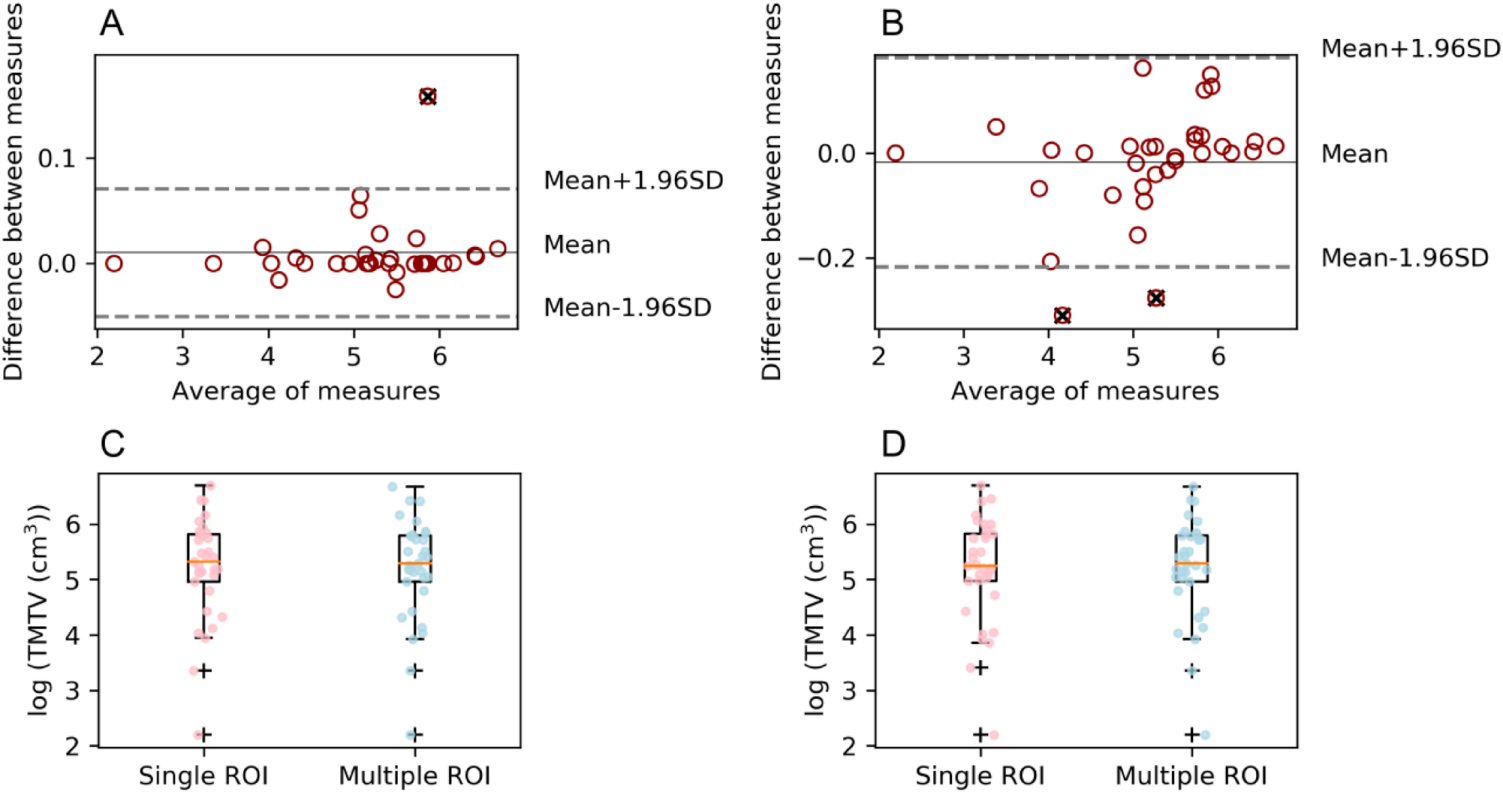
Multiple ROI study for the SUV > 2.5 threshold. Bland Altman analysis and Box Plot displaying the comparison of TMTV obtained using single ROI in BI and sum of multiple ROIs in BI (**A**, **C**) and LIFEx single ROI vs sum of multiple ROIs in BI (**B**, **D**).

For the 2.5 cut-off, more significant differences between single and multiple procedures were found for BI (r = 0.9994, p = 0.07) than for LIFEx (r = 0.995, p = 0.33). Using BI, for 41% (r = 0.94, p = 0.04) and 50% (r = 0.98, p = 0.007) statistical significance between compared values was obtained, as well as for LIFEx (r = 0.92, p = 0.0004, and r = 0.96, p < 10^–5^, respectively). The highest correlations were found between LIFEx single ROI and BI multiple ROI (Fig. 3), showing the effect of the use of multiple ROI drawings in BI compared to single ROI drawings in both BI and LIFEx, respectively.

**Figure 4:**
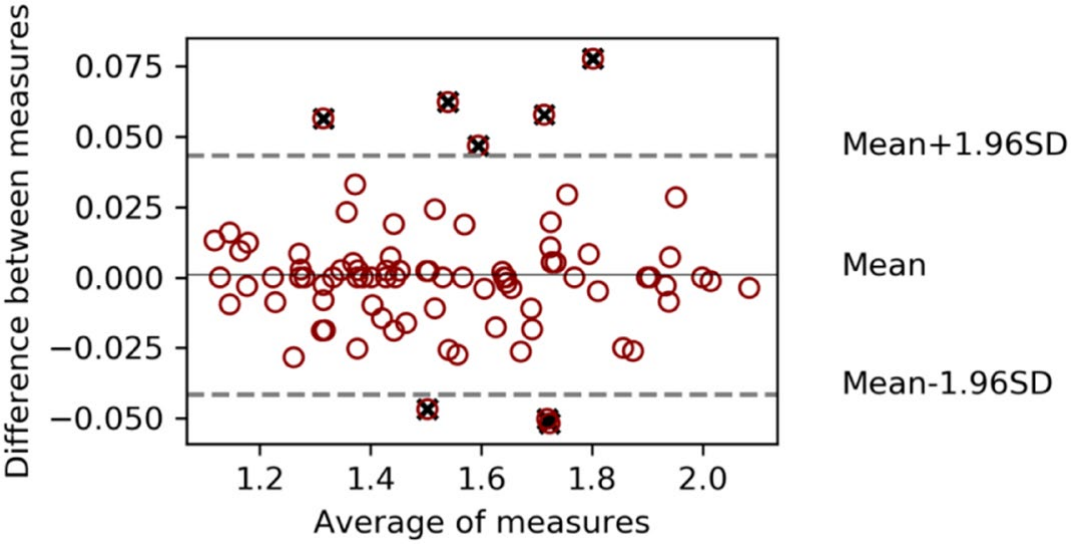
SUV_mean_ comparison using both software applying 2.5 SUV threshold. Limits of agreement are also displayed, as well as outliers marked with a black cross.

When comparing SUV_mean_ values obtained with BI and LIFEx, excellent correlation between them was found for the 2.5 cut-off (r = 0.996, p = 0.70), as well as for 41% (r = 0.97, p = 0.005) and 50% (r = 0.96, p = 0.02). No significant differences were observed in the analysis of the 2.5 cut-offs (Fig. 4).

**Figure 5:**
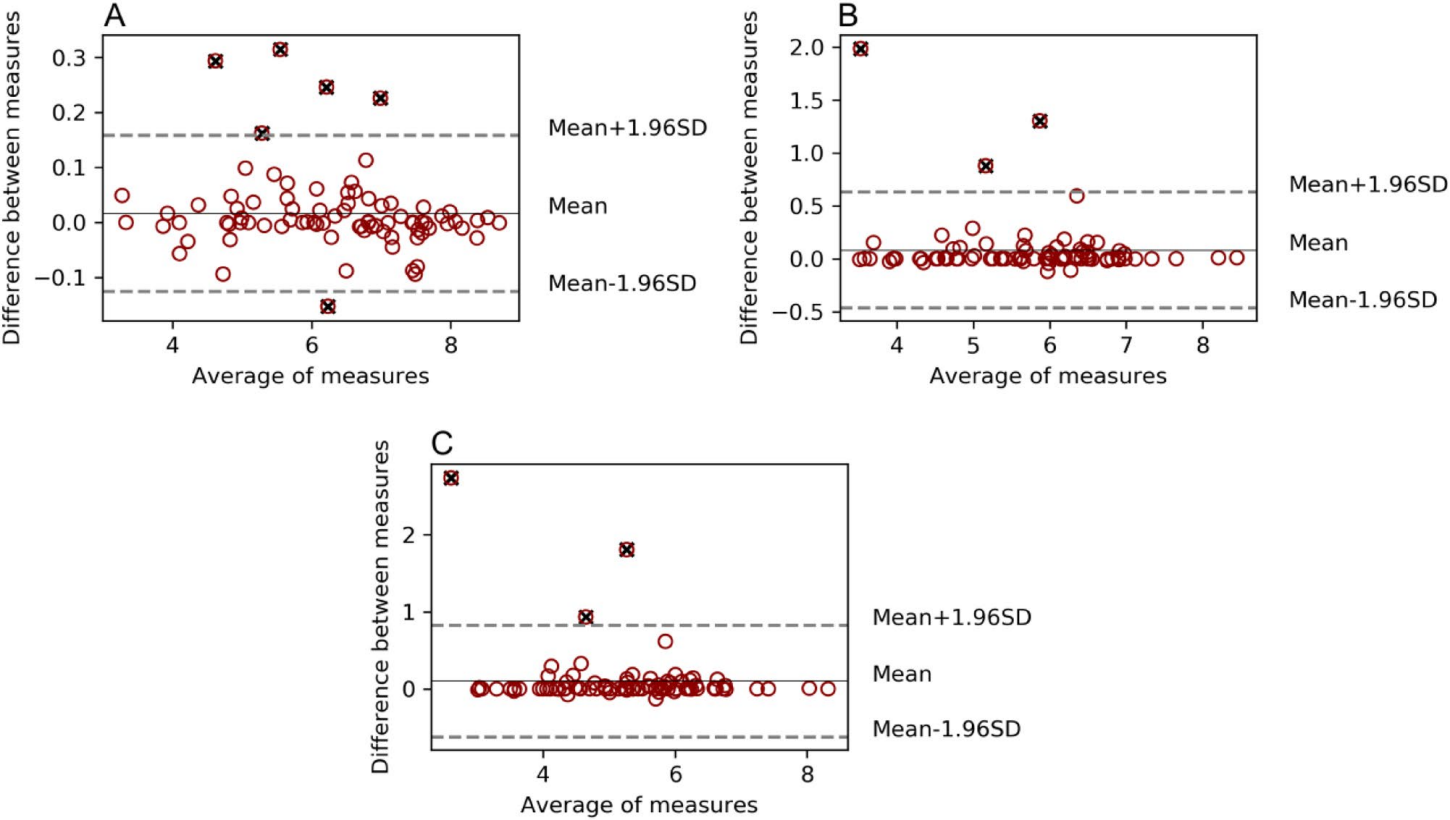
TLG Bland Altman analysis for SUV > 2.5 (**A**), SUV > 41% SUVmax (**B**), and SUV > 50% SUVmax (**C**).

The mean and median TLG values for BI were TLG_2.5_ 1,073.64 and 694.47 cm^3^ (range 27–6,028), TLG_41%_ 522.81 and 327.93 cm^3^ (range 34–4,689), and TLG_50%_ 380.86 and 214.80 cm^3^ (range 20–4,095). For LIFEx these values were 1,071.39 and 670.52 cm^3^ (range 26–6,031), 499.69 and 289.78 cm^3^ (range 13–4,643), and 363.88 and 211.13 cm^3^ (range 3–4,095), respectively. Similarities between both software could be found according to the studied cut-offs, TLG_2.5_ showed a slightly higher correlation (r = 0.9998, p = 0.03) than TLG_41%_ (r = 0.97, p = 0.008) and TLG_50%_ (r = 0.95, p = 0.015) (Fig. 5).

### Progression-free survival

**Figure 6:**
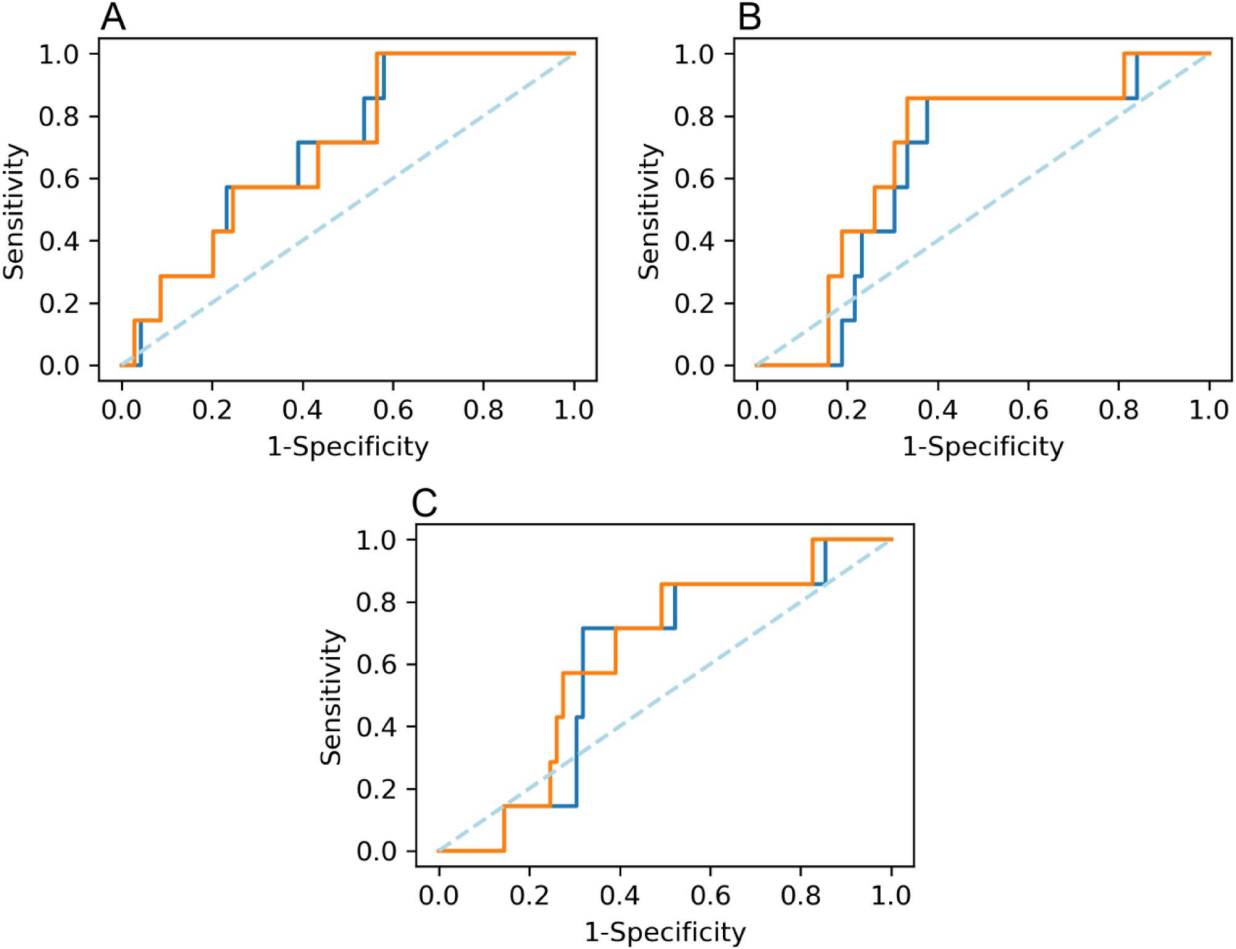
ROC curves comparing both software tools when using each threshold. Strong similarities are shown in 2.5 threshold. BI vs LIFEx 2.5 (**A**), BI vs LIFEx 41% (**B**), and BI vs LIFEx 50% (**C**). BI is displayed in blue and LIFEx in orange.

Optimal TMTV cut-offs found in ROC curves using 2.5, 41% and 50% thresholds for BI and LIFEx were 101.71 cm^3^ and 101.30 cm^3^, 63.18 cm^3^ and 59.07 cm^3^, and 41.86 cm^3^ and 26.41 cm^3^, respectively. More similarities were found between both software tools using 2.5 SUV threshold (Fig. 6).

AUC values for BI according to the mentioned thresholds (2.5, 41% and 50%) were 0.70, 0.64 and 0.60. For LIFEx obtained AUC were 0.70, 0.68 and 0.62, respectively. Discrimination performed by the model was acceptable: around 70% of the patients were correctly classified.

Highest AUC was obtained for the 2.5 threshold when comparing both software. No differences in prognosis were observed in none of them.

**Figure 7:**
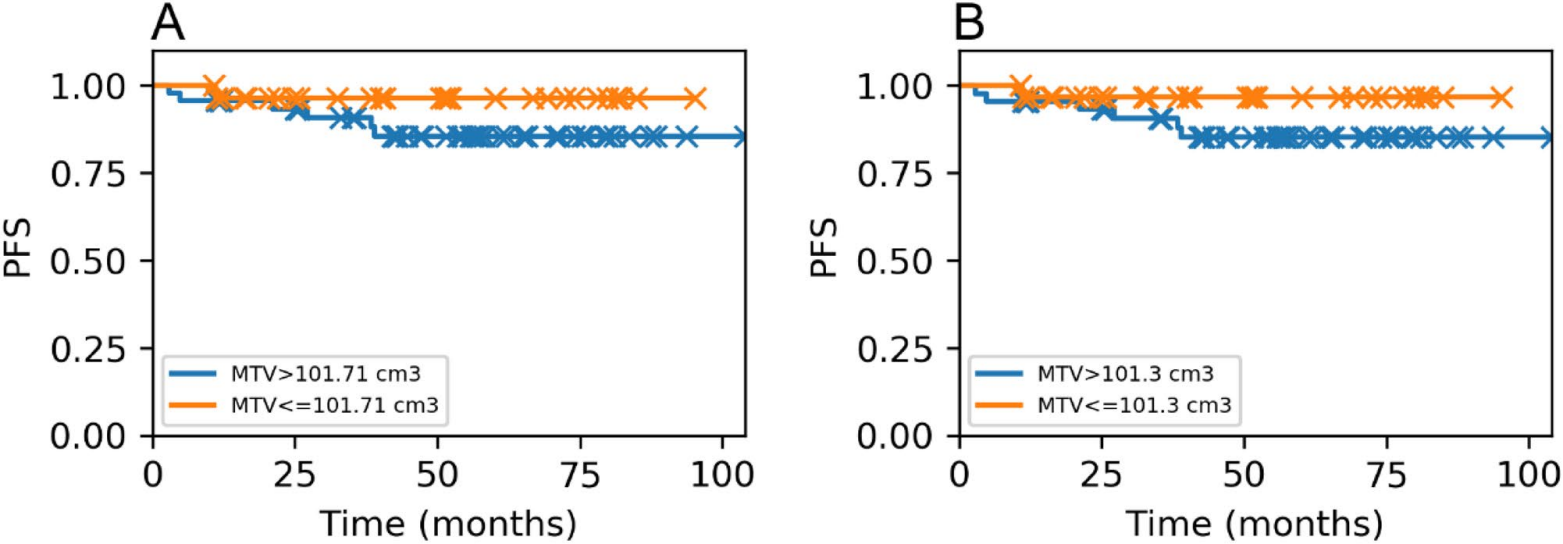
Kaplan–Meier analysis showing the predicted patient outcomes according to the TMTV cut-offs found in ROC for a SUV_2.5_ threshold in (**A**) BI and in (**B**) LIFEx.

PFS showed similarities between TMTV discriminated values for BI and LIFEx for all considered thresholds (2.5 p = 0.11 and p = 0.010, 41% p = 0.12 and p = 0.09, and 50% p = 0.25 and p = 0.26) (Fig. 7).

However, despite observing visually distinct curves, no significant differences were found between them due to the low number of patients with a TMTV bigger than the cut-off. Moreover, some patients did not have long enough follow-up, which could affect the behavior of such curves.

## Discussion

Response-adapted therapy using [^18^F]FDG PET/CT imaging at baseline (early detection) and interim has been proven to be a valuable predictor for disease prognosis in lymphoma patients^2,10,11^. The implementation of such methodology could be useful to avoid overtreatment in patients whose chemotherapy results in long-term PFS, as well as overall survival (OS)^12^. This could also help medical institutions to reach optimal patient management with customized treatments. SUV-based assessment on [^18^F]FDG PET/CT images has been shown to improve the prediction of patient outcome compared to visual analysis, due to interim PET false-positive results in HL^3^. However, no general agreement on the choice of threshold and software tools has been reached, meaning inter-institutional comparisons are hard to develop and no consensus on common clinical protocol has been attained. Therefore, different methodologies have been studied, using fixed and relative thresholds and different software tools to achieve successful TMTV delimitation in HL patients. Comparisons between them have been performed to find the best reproducibility between TMTV calculations; the effect of potential sources of discrepancy in patient outcome prediction has also been analyzed.

We restricted ourselves to localized lymphoma since we aimed to take a relatively homogeneous sample to avoid excessive multiple data correlation due to the heterogeneity in localized and advanced stage patients. If stage III–IV had been also included, a much extensive number of variables and considerations would have been needed. Non-localized lymphoma involves lesions which cannot be segmented using a regular threshold. The effect of different treatment regimens should be explored in future studies.

Although multiple physician supervision was not studied in the present work, as we relied in automatic methods for localized lesions, further research should be conducted on physician reproducibility especially in non-localized lymphoma.

In the present study, SUV_2.5_ thresholds overestimate TMTV and SUV values due to its segmentation simplicity. This statement is based mostly on visual assessment of the affected region compared to the one selected by the threshold. In many of the analysed patients the 2.5 threshold chose a wider region as lymphoma than expected, including some parts which were not affected by the disease. Absolute thresholds such as 2.5 tend to miss information from background activity and select voxels with intensity over a fixed value regardless of the lesion-to-background ratio, meaning that if voxels in lesion contour have a similar uptake to background voxels, these will be likely considered as a tumoral region.

The relative threshold approach is defined by assuming that those voxels having an intensity of at least a designated percentage of SUV_max_ will be part of TMTV^13^. High lesion-to-background ratio cases result in precise tumor delimitation. However, in patients whose lesions have low intensity, usage of relative thresholds can lead to overestimation due to a systematic lack of distinction between lesion and background detections. Despite that, if local uptakes inside the TMTV that are significantly higher compared to the rest of the metabolic active region are present, underestimation of the TMTV will have a high chance of occurring.

The 41% SUV_max_ threshold allows a much wider range of uptake values to be detected than 50% SUV_max_ when high intensities are being considered. The second mentioned cut-off misses most of the TMTV and only takes the surroundings of the region where SUV_max_ is found. Nonetheless, when SUV_max_ is not remarkably high, differences between both thresholds are only significant in bulky tumors.

Contouring was based on delimiting the ROI that included as many tumoral uptakes as possible and avoided all physiological detections. ROI drawing depended mostly on the software tool. BI had more limitations in the drawing process because the region was chosen on a single slice and then extrapolated to the limiting slices; limits were displayed in z-axis for the axial view. Those limitations were due to the software tool as the drawing of our region was performed in only one of the slices which implied that in some of the cases physiological uptake areas were incorrectly detected as disease. This issue affected the correct discrimination of tumoral areas in some of the patients where lesions laid close to healthy organs with [^18^F]FDG uptake. In these situations, the use of multiple ROI to delimit tumoral areas avoided the misdetection of physiological activity. Compared to LIFEx, this was no longer an issue because of the precise painting tools available for contouring and the existence of a region eraser for those areas that had been mistakenly chosen as lymphoma. Drawing was therefore more precise but slower to perform since ROI delimitation was modified slice by slice. To obtain the first indicative delimitation of the region in BI took us approximately 10 min, while for LIFEx implied a minimum of 15 min and up to 30 min. This is displayed as an average of the employed time, but it also depended on the closeness of lesions to physiological uptakes and the extensivity of the affected region. Since the data set included 76 patients, the difference of time required to perform the contouring between both software was large enough to consider LIFEx a slower method. Maximum accuracy for contouring took more time but oscillated depending on the patient, especially for multiple lesion affected ones; however, all healthy organs could be avoided using a single ROI since physiological detections were scarcely displayed in images.

Finding the best methodology would mean achieving exceptional reproducibility between TMTV values, as well as finding similarities between SUV parameters and TLG, which are good indicators of tumor activity.

In the single ROI study, software comparison between BI and LIFEx showed good reproducibility for TMTV_2.5_ compared to TMTV_41%_ and TMTV_50%_ due to the use of the same segmentation criteria and the absence of algorithm complexity. However, 41% and 50% thresholds showed similarities within the software in single ROI studies. 41% and 50% SUV_max_ thresholds showed an excellent correlation between them when using the same software for both BI and LIFEx; this was due to BI single ROI mistaken delimitation which overestimated the tumoral region when using SUV_2.5_ threshold that differed from the TMTV obtained with 41% and 50%.

Multiple ROI study showed a better correlation between LIFEx single and BI multiple than LIFEx single and BI single. Moreover, when comparing LIFEx single and BI multiple with BI single and BI multiple, a stronger correlation was found between different software tools. This could be due to the fact that BI single ROI drawing had mistakenly taken healthy tissue as the tumoral region in some patients where lesions were close to physiological detections. LIFEx single contouring and BI multiple ROI drawing did not take these as part of the TMTV, which resulted in reliable TMTV delimitation. Therefore, the best reproducibility of TMTV values in all performed studies was found for LIFEx single ROI and BI multiple ROI drawing, regardless of the applied threshold. Reproducibility was tested comparing the results obtained using different methodologies including software tool, threshold and multiple or single ROI study choice. High reproducibility was considered for those studies which showed strong similarities between the obtained values, basing our criteria on the ability of replicating the data obtained under different methods.

The SUV_mean_ values differed between thresholds within the same software tool but took similar values in 41% and 50% comparison. A strong correlation was found between obtained values using different software. Hence, the comparison of calculated TLG values using the same threshold showed a high correlation as well. However, TMTV_2.5_ having the best reproducibility resulted in the strongest correlation for TLG_2.5_ in software comparison.

The SUV_2.5_ threshold showed higher values for TMTV and SUV parameters, as well as for TLG calculation. This was due to the mentioned overestimation intrinsic to the cut-off caused by arbitrary segmentation algorithms. Consequently, optimal metabolic tumor volume predicted by ROC curves for 2.5 was also greater than the obtained using 41% and 50% thresholds. Nevertheless, measured differences in TMTV did not show an impact on computed survival curves.

Similarly Cottereau et al.^14^ compared different adaptive thresholds to the SUV_max_ based ones, and concluded that the definition of the TMTV is not crucial to obtain an initial good prognostic value for peripheral T cell lymphoma. Our results are consistent with the idea that an accurate definition of the TMTV is not as important as other factors in general case mixing different stages and treatment protocols.

Furthermore, in previous studies Kanoun et al.^6^ used 2.5, 41%, 125% liver SUV_max_ and 140% liver SUV_max_, and found optimal cut-offs for prediction of treatment failure according to the mentioned thresholds and, finding cut-offs and AUC values for 2.5 and 41% higher than those obtained in our study. However, patient selection can affect TMTV depending on the Ann-Arbor stage. Kanoun et al. considered stages I to IV. Song et al. performed their study with patients at stages I–II and their optimal cut-offs using 2.5 threshold were only a bit higher than the obtained in our study using 2.5^11^. Another factor influencing these discrepancies could be the number of patients with bulky tumors, which would lead to higher TMTV detection; Song et al. had 26 bulky patients while we only accounted 7.

Regarding our results, further work should be done assessing the impact of different acquisition settings and machinery choice, as there could be an impact on MTV measurements.

Our study did not consider the use of adaptive algorithms in TMTV discrimination. Only fixed and relative thresholds available within the software tool were analyzed. Erdi et al. found that adaptive thresholds worked optimally for small tumors. For lesions > 4 cm^3^, image segmentation converged to fixed thresholds from 36 to 44%^15^. Patient database only included HL individuals from our institution. Also, we did not include modern algorithms as Q.Clear which could have a bigger impact on the delimitation of the TMTV^16,17^. Forthcoming studies including patient sets from different medical centers could allow a deeper analysis of distinct methodologies, which could lead to a future unification of TMTV delimitation method.

## Conclusions

Unification of methodologies in TMTV delimitation needs to be achieved in order to improve patient management in medical centers despite the absence of differences in survival prediction regarding each studied method.

Our study suggests that TMTV based on SUV 2.5 threshold is the most reproducible and robust parameter under the choice of a software tool or delimitation method; the overestimation of TMTV seems to be desirable rather than underestimation in terms of reproducibility. Best reproducibility regarding software tools was found for LIFEx single ROI and for BI multiple ROI drawing, regardless of the applied threshold.
